# Effect of Cotton Cationization Using Copolymer Nanospheres on Ink-Jet Printing of Different Fabrics

**DOI:** 10.3390/polym10111219

**Published:** 2018-11-02

**Authors:** Haizhen Yang, Kuanjun Fang, Xiuming Liu, Yuqing Cai, Fangfang An

**Affiliations:** 1 School of Textiles, Tianjin Polytechnic University, No. 399 Binshui Xi Road, Xiqing District, Tianjin 300387, China; 13702189153@163.com (H.Y.); yushimylove@163.com (X.L.); 13933186174@163.com (F.A.); 2 Collaborative Innovation Center for Eco-Textiles of Shandong Province, No. 308 Ningxia Road, Qingdao 266071, China; 3 School of Textiles and Clothing, Qingdao University, No. 308 Ningxia Road, Qingdao 266071, China; 13954216129@163.com

**Keywords:** ink-jet printing, cationization, reactive dyes, color strength, outline sharpness

## Abstract

In this study, the cationic Poly[Styrene-Butyl acrylate-(P-vinylbenzyl trimethyl ammonium chloride)] P(St-BA-VBT) nanospheres with N^+^(CH_3_)_3_ functional groups were successfully synthesized by soap-free emulsion polymerization and applied to different fabrics by pad-cure process. After the pad-cure process, the nanospheres were deposited on the surface of the modified cotton fibers successfully without forming a continuous film structure. The X-ray Photoelectron Spectroscopy (XPS) and the Fourier transform infrared (FTIR) results demonstrated that P(St-BA-VBT) nanospheres were adsorbed on the surface of cotton fibers successfully. The excellent color strength value and outline sharpness of the plain, twill, and honeycomb fabrics can be achieved when the nanosphere concentration, sodium bicarbonate, and steaming time were 1 g/L, 10 g/L, and 6 min, respectively. The plain fabrics exhibited the smallest color strength (*K*/*S*) values and the best outline sharpness, followed by twill and honeycomb fabrics, which displayed the largest *K*/*S* values and the worst outline sharpness after the same treatment. Besides, all the three fabrics showed excellent rubbing fastness and washing fastness. The cationic P(St-BA-VBT) nanospheres modification of the cotton fabrics provides a novel potential approach to obtain good printing efficiency without affecting the tensile breaking strength of cotton fabrics significantly.

## 1. Introduction

Ink-jet printing of textiles has become increasingly important and popular in the textile industry due to its higher quality image and lower resource consumption and wastewater discharge compared to conventional printing [1,2,3,4]. However, surface pretreatment of the fabrics must be conducted before the ink-jet printing with reactive dyes to increase the printing precision and the dye utility [5,6,7,8]. Conventional printing thickeners such as sodium alginate, carboxyl methyl cellulose, and acrylate copolymers are usually used to pretreat the fabrics. However, the thickeners, urea, and chemicals padded on the fabrics must be washed off after steaming of the printed fabrics in order to improve the handle and the color effect and to remove the hazardous chemicals. This not only increases the water and energy consumption, but also increases the pollutant contents of wastewater.

The function of fabric surface pretreatment is to immobilize the ink drop jetted onto the fabric to prevent its bleeding. In order to solve the environmental problems caused by using printing thickeners to pretreat fabrics for ink-jet printing, plasma technology and cationic compounds have been used to pretreat the fabrics [9,10]. Silk fabrics pretreated with oxygen plasma enhance the ink-jet printing image quality [11]. Air plasma has also been used to pretreat polyester fabrics to improve the pigment ink-jet printing effect [12]. Cotton fabrics cationized with 3-chloro-2-hydroxypropyltrimeth-ylammonium chloride solutions and then pretreated with a medium viscosity alginate thickener showed a very higher color strength and pattern sharpness compared with the non-cationized fabrics [13,14]. Surface modification of cotton fabrics using cationic nanospheres was proved to possess strong electrostatic attraction to the negatively charged dyes to increase the dye utility and fabric handle and to obtain a salt-free dyeing effect [15,16]. For example, cotton fabrics modified with cationic copolymer nanospheres were dyed by an acid dye, revealing that the dyed fabrics had good handle and permeability [17]. Cotton fabrics pretreated with Poly[Styrene-Butyl acrylate-Acrylic acid-Glycidyl methacrylate] P(St-BA-AA-GMA) nanoparticles had better pigment dyeing properties than the fabrics pretreated with Poly[Styrene-Butyl acrylate-Acrylic acid] P(St-BA-AA) nanoparticles [18]. Fabrics modified with the cationic nanospheres displayed the advantages of high dye utilization and excellent color performance [19,20,21].

Due to the dye molecules in the ink drop being ionized, the ink drops are negatively charged. Therefore, the ink drops could be immobilized on the cationized fabric surface. The objective of the present study aimed to explore the ink-jet printing effect of cotton fabrics pretreated by cationic Poly[Styrene-Butyl acrylate-(P-vinylbenzyl trimethyl ammonium chloride)] P(St-BA-VBT) nanospheres to reduce the pollutant contents in the wastewater. We prepared the cationic copolymer nanospheres dispersion by soap-free emulsion polymerization. Three kinds of cotton fabrics, plain, twill, and honeycomb fabrics, were pretreated using the synthesized nanospheres dispersion to investigate the effect of nanospheres pretreatment on differently textured fabrics. The color performance of ink-jet printed fabrics was analyzed. The results show that the cationic polymer microspheres pretreatment significantly enhanced the color performance of ink-jet printed fabrics. More importantly, the nanospheres pretreated on the fabrics were fixed on the fiber surfaces, and the pretreatment avoided the use of urea. Therefore, the pollutant contents in the wastewater were evidently reduced compared with the conventional thickener pretreatment, meaning that the reported process is environmental friendly.

## 2. Materials and Methods 

### 2.1. Materials

#### 2.1.1. Textile Material

**Figure 1:**
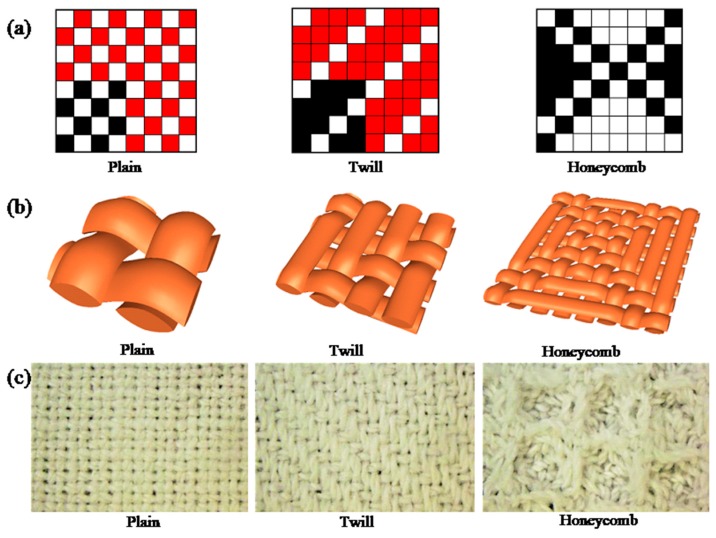
Three types of woven fabrics shown on the weaving paper (**a**), three-dimensional (**b**), and fabric photographs (**c**).

The cotton fabrics used were de-sized and scoured standard (plain weave, yarn count: warp 180 ends/10 cm, weft 150 ends/10 cm), (3/1 twill weave, yarn count: warp 180 ends/10 cm, weft 150 ends/10 cm), (honeycomb weave, yarn count: warp 180 ends/10 cm, weft 150 ends/10 cm), woven by ASL2300 automatic rapier loom (Tianjin Longda Electromechanical Technology Development Co., Ltd., Tianjin, China). Three types of woven fabrics were shown on the weaving paper, three-dimensional and fabric photographs were illustrated in Figure 1. Roughness, thickness and porosity of fabrics with different structures were displayed in Table 1.

#### 2.1.2. Chemical and Reagents

Styrene (St) was supplied by Tianjin Ruijinte Chemical Reagent Co., Ltd., Tianjin, China. Butyl acrylate (BA) was provided by Tianjin BASF Chemical Co., Ltd., Tianjin, China. P-vinylbenzyl trimethyl ammonium chloride (VBT) was purchased from Tianjin Heowns Biochem Technologies (Tianjin, China). 2,2′-azobis [2-methylpropionamidine] dihydrochloride (AIBA) was provided by Qingdao Kexin New Material Technology Co., Ltd., Qingdao, China. Analytical grade of sodium bicarbonate was obtained from Tianjin Kemiou Chemical Reagent Co., Ltd., Tianjin, China. The cyan reactive dye inks were purchased from Hangzhou Honghua Digital Technology Co., Ltd., Hangzhou, China. All the reagents were used as received without further treatment. The deionized water was used throughout the experiments.

### 2.2. Preparation of Cationic P(St-BA-VBT) Nanospheres

**Figure 2:**
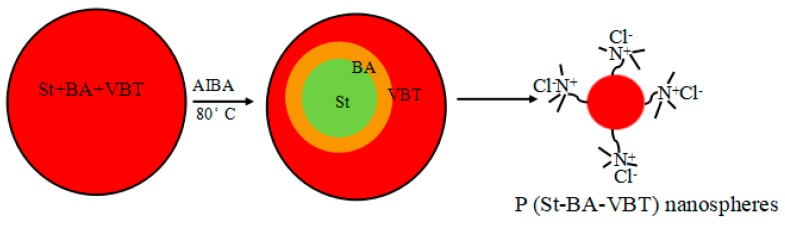
The polymerization for preparing the cationic P(St-BA-VBT) nanospheres.

The cationic P(St-BA-VBT) nanospheres were prepared according to Guo et al. [22]. The deionized water (90 mL) was firstly added to a 250 mL four-necked flask equipped with a stirrer, a condenser, and a nitrogen inlet. After 5 min of nitrogen gas, the VBT solution (5 mL, 0.4 mol/L) was added into the four-necked flask and stirred for 15 min at the stirring speed of 300 r/min, then the solution mix with St (9.2 g) and BA (0.8 g) was added and stirred for 2 h. Subsequently, the temperature of the system was raised from 25 °C to 80 °C, the AIBA (5 mL, 20 g/L) solution was added in 5 min and reacted at 80 °C for 4 h. Finally, the materials were cooled and the P(St-BA-VBT) nanospheres were obtained. The polymerization for preparing the cationic nanospheres was illustrated in Figure 2.

### 2.3. Cationization Modification of Cotton Fabrics

The cationic P(St-BA-VBT) nanospheres modification of cotton fabrics was carried out according to the Pad-Batch method [15]. The concentration of the P(St-BA-VBT) nanospheres changed from 0.5 g/L to 2.5 g/L, and the concentration of sodium bicarbonate changed from 1 g/L to 25 g/L. The fabrics were padded through the cationization modification fluid with a pick-up of (70 ± 1)%, and then fabrics were dried at 80 °C for 5 min in a DHG-9123A oven.

### 2.4. Ink-jet Printing of Cotton Fabrics

The ink-jet printing was carried out on a digital ink-jet printer VEGA 5000 (Hangzhou Honghua Digital Technology Co., Ltd., Hangzhou, China) at the resolution of 720 × 720 dpi by applying cyan reactive dye inks. One 100% color filled rectangle pattern of 80 × 150 mm and lines of 1 mm were printed on the fabrics. Subsequently, all fabrics were dried at 95 °C for 5 min and then steamed in a steamer STM-G2003 (Suzhou Industrial Park YAMEI Textile Machine Co., Ltd., Suzhou, China) at (102 ± 1) °C for 1–16 min. After steaming and fixing, all fabrics were firstly washed with cold water, and then washed by hot water with 2 g/L soap flakes, and followed by washing with warm water until no color could be removed from the fabrics and drying at 80 °C for 5 min.

### 2.5. Measurements

#### 2.5.1. Transmission Electron Microscopy (TEM) Analysis

The cationic P(St-BA-VBT) nanosphere samples were ultrasonically diluted 25 times with deionized water by a Bilon-1000 ultrasonic signal transmitter (Bilang Instruments Co., Ltd., Shanghai, China) and were dropped onto Cu meshes. After drying under a 701 infrared lamp (Tianyu Experimental Instrument Co., Ltd., Tianjin, China), the sizes and morphologies of the cationic P(St-BA-VBT) nanospheres were observed using a H7650 transmission electron microscope (Hitachi, Tokyo, Japan). The diameters of 100 different nanospheres were measured from the TEM images. The average diameter *D* of the nanospheres was calculated according to the Equation (1):

(1) $$D=\sum_{i=1}^{n}d_{i}/n$$

where *n* equals 100, and *d_i_* is the diameter of nanosphere *i*.

#### 2.5.2. Sizes and Zeta Potential of the Nanospheres

The hydrodynamic particle size of P(St-BA-VBT) nanospheres was measured by a dynamic light scattering instrument (Malvern Nano-Zs90 nano-particle size analyzer, Malvern Worcestershire, UK). The Zeta potential was determined by a Zeta potential analyzer (Malvern, Malvern Worcestershire, UK). A certain amount of P(St-BA-VBT) nanospheres samples were diluted 2000-fold with deionized water before test.

#### 2.5.3. Differential Scanning Calorimetry (DSC) Analysis

The glass transition temperature of P(St-BA-VBT) nanospheres was measured by a differential scanning calorimetry (Netzsch DSC 204F1, Netzsch Group, Bavaria, Germany) under nitrogen (20 mL/min) atmosphere at 10 °C/min from 20 °C to 200 °C [18].

#### 2.5.4. Colorimetric Data and Outline Sharpness of Ink-jet Printed Fabrics

The color strength (*K*/*S* value) and CIE L*, a*, b*, C*, h* of ink-jet printed fabrics were measured by a Datacolor SF-600 plus (Datacolor Co., Lawrenceville, NJ, USA) with D_65_ illumination and 10° standard observer [23]. The *K*/*S* value was assessed at the maximum absorption wavelength of 670 nm. Each fabric was folded into four layers and measured at ten different locations. The average *K*/*S* value was assessed based on the Kubelka Munk Equation (2):

(2) $$K/S={\left(1-R\right)}^{2}/2R$$

where *R* is reflectance of ink-jet printed fabrics at maximum absorption wave length, *K* and *S* are absorption coefficient and scattering coefficient of ink-jet printed fabrics, respectively. The colors are given in CIELAB coordinate system: L* presents luminosity, a* presents redness-greenness (+value = red, −value = green), b* presents yellowness-blueness (+value = yellow, −value = blue), C* presents saturation, and h* presents metric hue angle [24,25].

The outline sharpness of ink-jet printed fabrics was measured by a YYS-560E optical microscope (Shanghai Yiyuan Optical Instrument Co., Ltd., Shanghai, China) with a magnification of 40×.

#### 2.5.5. Rubbing and Washing Fastness

Dry and wet rubbing fastness properties of ink-jet printed cotton fabrics were tested according to ISO 105-X12: 2001 using the Y571 rubbing machine (Nantong Hongda Experiment Instruments Co., Ltd., Nantong, China). The washing fastness was measured according to the methods established in ISO 105-C10:2007 using the SW-24 washing colorfastness tester (Laizhou Yuanmao Instrument, Co., Ltd., Laizhou, China).

#### 2.5.6. Scanning Electron Microscopy (SEM) Analysis

The cotton fabric was firstly immersed the modification solution (containing 1 g/L of nanospheres and 10 g/L of sodium bicarbonate) and then passed through a padding mangle to obtain (70 ± 1)% pickup. The padded sample was dried at 80 °C for 5 min. The cotton fabric obtained was denoted as treated cotton fabric. The surface morphology of untreated and treated fabrics was observed by a Hitachi S-4800 field emission scanning electron microscope (Hitachi, Tokyo, Japan) operating at 10 kV; all samples were coated with a thin layer of gold before the observation.

#### 2.5.7. X-ray Photoelectron Spectroscopy (XPS) Analysis

The chemical compositions of both the untreated and treated cotton fabrics were analyzed using a K-Alpha X-ray photoelectron spectrometer (Thermo Fisher Scientific Co., Ltd., Waltham, MA, USA) with an Al K-Alpha source type at an incident energy of 1486.6 eV. For analyzing the general spectra, the spot size, pass energy and energy step size were separately set as 400 µm, 200.0 eV and 1.0 eV. All measurements were made at an UHV chamber pressure between 5 × 10^−9^ and 2 × 10^−8^ Torr. The C1s peak was referred to a C–C binding energy of 285.0 eV [19].

#### 2.5.8. Fourier Transform Infrared (FTIR) Analysis

The Fourier transform infrared spectroscopy spectrums of the untreated and treated cotton fabrics were conducted by a TENSPR37 spectrometer (Bruker Co., Ltd., Hamburg, Germany). Testing the infrared range was logged in 600–4000 cm^−1^ interval with 1 cm^−1^ resolution.

#### 2.5.9. Tensile Breaking Strength

The breaking strength of cotton fabrics was measured by the YG065 electronic fabric strength tester (Laizhou Electron Instrument Co., Ltd., Laizhou, China) according to the method of GB/T 3923.1–2013: Textiles-tensile properties of fabrics-part 1. The effective length and width of the fabrics were 250 and 50 mm, respectively. The tensile velocity was set as 100 mm/min at room temperature. Each fabric sample was tested three times to calculate the average measurement.

## 3. Results and Discussion

### 3.1. Properties of Cationic P(St-BA-VBT) Nanospheres

**Figure 3:**
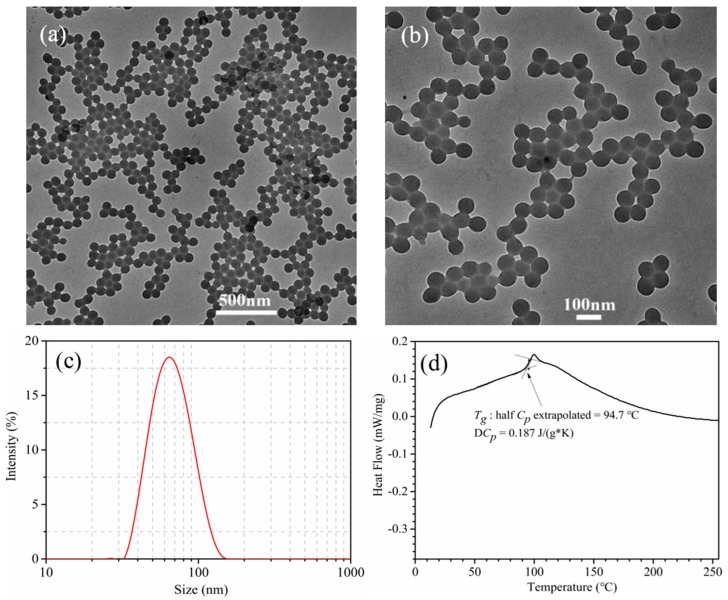
TEM images of P(St-BA-VBT) nanospheres with low magnifications (**a**), high magnifications (**b**), size distributions of P(St-BA-VBT) nanospheres (**c**), and differential scanning calorimetry (DSC) curve of P(St-BA-VBT) nanospheres (**d**).

Figure 3a,b represent the TEM images of P(St-BA-VBT) nanospheres with different magnifications. It was obvious that P(St-BA-VBT) nanospheres synthesized by the soap-free emulsion polymerization method exhibited a regular spherical shape with smooth surface, uniform particle size, and good dispersion. Figure 3c exhibits the sizes and distributions of the nanospheres. It was apparent that most of the nanospheres displayed a diameter less than 100 nm, the average diameter of the nanospheres was 65.5 nm. In addition, the prepared nanospheres had a zeta potential of +57.8 mV due to the existence of cationic groups of cationic initiators and cationic emulsifiers during the synthesis of cationic nanospheres [26,27].

The DSC curve of the P(St-BA-VBT) nanospheres was illustrated in Figure 3d. Obviously, the glass transition temperature (*T*g) of the nanospheres was 94.7 °C, indicating that the nanospheres could be applied to the cationization modification of cotton fabrics and avoid the uneven ink-jet printing phenomenon of cotton fabric [28].

### 3.2. Influence of Cationic Nanospheres on K/S Value and Outline Sharpness of Ink-jet Printed Cotton Fabric 

**Figure 4:**
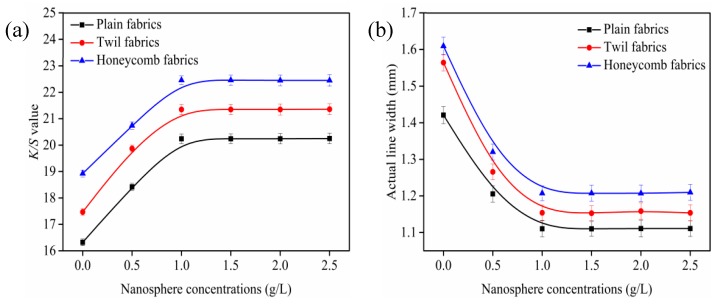
Effect of cationic nanospheres on color strength (*K*/*S*) values (**a**) and outline sharpness (**b**), fabric was printed with cyan as a rectangle pattern of 80 × 150 mm and lines of 1 mm. All the pickup of fabrics were 70 ± 1% and steaming temperature was 102 ± 1 °C.

The changes of *K*/*S* value and outline sharpness of ink-jet printed cotton fabrics with different concentrations of cationic nanospheres were subjected in Figure 4.

As shown in Figure 4a, the *K*/*S* value of ink-jet printed fabrics increased rapidly with the concentration of cationic nanospheres increased from 0 to 1.0 g/L, and then leveled off when the concentration of cationic nanospheres exceeded 1.0 g/L. The results indicated that the higher the nanosphere concentration, the darker the color strength of ink-jet printed fabrics, which can be ascribed to the increasing positive charge on the fabric surfaces. For the reason that the positive charge on the cotton fiber surfaces were introduced owing to the quaternary ammonium groups of cationic nanospheres, and hence the cotton fiber affinity to attract the anionic reactive dye molecules increased. Therefore, the reactive dye molecules not only reacted with the cotton hydroxyl groups but also reacted with the quaternary ammonium groups of the nanospheres in the ink-jet printing process, resulting in the increase *K*/*S* value of the ink-jet printed fabric. While when the concentration of nanospheres exceeded 1.0 g/L, the reactive dyes adsorbed on the cotton fibers had reached equilibrium and there were no more free and accessible sites for the dyes to attach, resulting in less changes of the *K*/*S* value. The maximum *K*/*S* value can be obtained when the concentration of cationic nanospheres was 1.0 g/L, which were 20.2, 21.4 and 22.5 for the plain, twill, and honeycomb fabrics, respectively.

Figure 4b presents the effect of cationic nanospheres on ink-jet printed outline sharpness. It can be seen that the actual line width of untreated plain, twill, and honeycomb fabrics is larger, indicating severe percolation and poor outline sharpness. It can be attributed to the less cohesion to the ink droplets of the untreated fabrics which made the ink droplets spread along the yarns [29,30]. The actual line width of ink-jet printed fabrics gradually decreased as the concentration of cationic nanospheres increased, indicating that the outline sharpness of ink-jet printed fabrics was improved. It can be explained by the positive charges on the surface of fibers introduced by the cationic nanospheres, which can strongly attract the anionic reactive dyes and hinder the further diffusion of the ink droplets on the fabric or fiber interior [31,32]. The actual line width of the three fabrics leveled off when the concentration of cationic nanospheres exceeded 1.0 g/L, which indicated that the 1.0 g/L concentration of cationic nanospheres was the optimal to obtain excellent outline sharpness for plain, twill and honeycomb fabrics. Therefore, the 1.0 g/L of nanospheres was selected in following investigation.

It can also be seen from Figure 4, the plain fabric exhibited the smallest *K*/*S* values and the best outline sharpness, followed by the twill fabric and the honeycomb fabric displayed the largest *K*/*S* values and the worst outline sharpness under the same nanosphere concentration conditions. Combined with Figure 4 and Table 1 analysis, the reason was that the warp and weft interlacing points of twill fabrics were less than those of plain fabrics, and the warp roughness, weft roughness, fabric thickness and porosity were larger than those of plain fabrics. The gaps between the yarns, fibers and fibrils made the inks more easily diffuse into the fabric interior and penetrate into the amorphous region of fibers with the help of the capillary pressure and hydrogen bonding, which contributed to most of reactive dyes being fixed on the fabric surface [33,34,35]. The color of ink-jet printed fabric is determined by the light source irradiation on the fabric and the light absorption, reflection, transmission, and scattering of the fabric and observer together [35]. The distribution of dye on the fabric has a great influence on light absorption and reflection. Therefore, the more dyes fixed on the fabric surface resulted in an increase in the absorption of light by the fabric and a decrease the reflectance of light. It can be seen from Equation (2) that the color strength value of fabrics increases as the reflectance decreases. For the same reason, this was also the reason why the *K*/*S* values and actual line width of honeycomb fabrics were higher than those of twill fabrics after the same treatment.

### 3.3. Influence of Steaming Time on K/S Value and Outline Sharpness of Ink-Jet Printed Cotton Fabric

**Figure 5:**
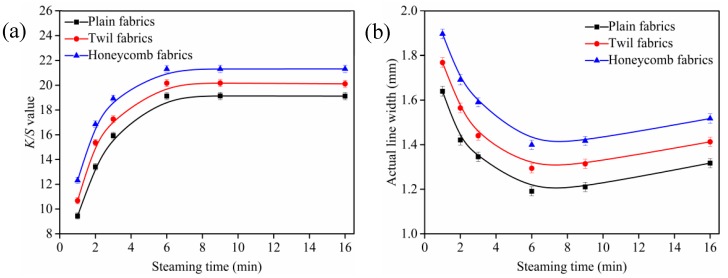
Effect of steaming time on *K*/*S* values (**a**) and outline sharpness (**b**), fabric was printed with cyan as a rectangle pattern of 80 × 150 mm and lines of 1 mm. All the pickup of fabrics were (70 ± 1)%, and steaming temperature was (102 ± 1) °C.

The plain, twill, and honeycomb fabrics were padded through modification fluid (1 g/L of nanospheres and 10 g/L of sodium bicarbonate) with a pick-up of 70 ± 1% and then steamed at 102 ± 1 °C from 1 min to 16 min. The *K*/*S* values and outline sharpness of ink-jet printed cotton fabrics with different steaming time are shown in Figure 5.

As shown in Figure 5a, the *K*/*S* values of ink-jet printed cotton fabrics increased rapidly with steaming time increased from 1 to 6 min, and then levelled off when the steaming time was larger than 6 min, which indicated that the nanosphere modification process of cotton fabric was completed in a short time. Meanwhile, some reactive dyes could be spontaneously adsorbed on the cationic cotton fibers through electrostatic attraction and penetrate into the fiber interior rapidly during the steaming process. In addition, the amount of nanospheres on cotton fibers surfaces had reached equilibrium and there were no more free and accessible sites for the reactive dyes to attach [36], resulting in less changes of the *K*/*S* values. The steaming time of 6 min for plain, twill and honeycomb fabrics modified with nanospheres was the optimal to obtain the product with excellent outline sharpness under the steaming condition studied.

Figure 5b illustrated the effects of steaming time on outline sharpness of ink-jet printed cotton fabrics. It was obvious that the actual line width of ink-jet printed cotton fabrics decreased first and then increased with the extension of the steaming time. As the steaming time increased, more dyes could penetrate into the fiber interior, resulting in a reduction in the actual line width and an increase in the outline sharpness of ink-jet printed fabrics. However, the reactive dyes adsorbed on the cotton fibers had reached equilibrium after 6 min and the excess dyes no longer penetrated into the fiber interior. On the contrary, the reactive dyes spread further along the yarns, resulting in an increase in the actual line width and a decrease in the outline sharpness [36]. Therefore, the optimal steaming time of plain, twill and honeycomb fabrics was 6 min, and it was selected in following investigation.

### 3.4. Influence of NaHCO_3_ Amounts on K/S Value of Ink-jet Printed Cotton Fabric

**Figure 6:**
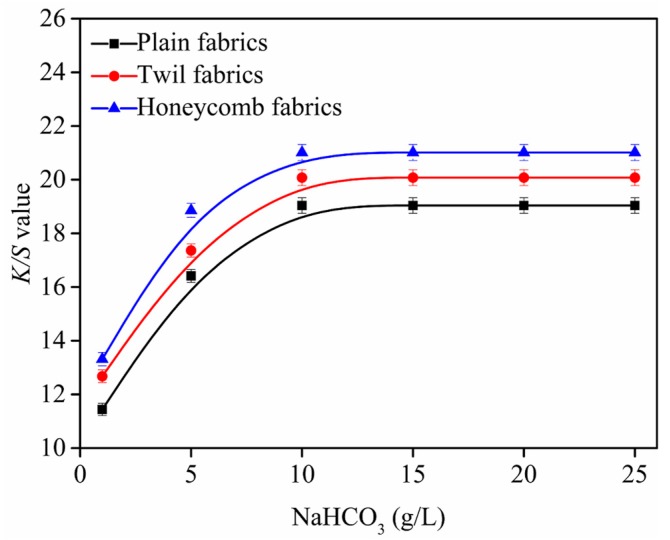
Effect of NaHCO_3_ on *K*/*S* value, fabric was printed with cyan as a rectangle pattern of 80 × 150 mm and lines of 1 mm. All the pickup of fabrics were 70 ± 1% and steaming temperature was 102 ± 1 °C.

The changes of *K*/*S* value of ink-jet printed cotton fabrics with various amounts of NaHCO_3_ are displayed in Figure 6.

As shown in Figure 6, it can be seen that the *K*/*S* values of ink-jet printed cotton fabrics gradually increase as the amounts of NaHCO_3_ increase from 1 g/L to 10 g/L, and the maximum *K*/*S* value can be achieved when NaHCO_3_ amounts achieve 10 g/L. It can be explained that the surface of fabrics become more alkaline with the amounts of NaHCO_3_ increases, which can quickly dissociate more hydroxyl groups on the fibers and enhance the degree of reaction with the reactive dyes [15,16], resulting in most of reactive dyes fixing on the fibers. For the cotton fabrics, when the amounts of NaHCO_3_ exceed 10 g/L, the *K*/*S* values displayed a downward trend, because the excess alkali increased the degree of hydrolysis of the reactive dye. In addition, excess alkali can damage cotton fibers [15,16]. Therefore, the NaHCO_3_ of 10 g/L was the optimal to obtain the product with good *K*/*S* values for plain, twill and honeycomb fabrics, and it was selected in following investigation.

### 3.5. Color Characteristic and Color Fastness of Cotton Fabrics

Table 2 illustrates the color characteristics of ink-jet printed cotton fabrics with different structures. All fabrics were padded through cationization modification fluid (1 g/L of nanospheres and 10 g/L of sodium bicarbonate) with a pick-up of 70 ± 1%. The colors are given in CIELAB coordinate system: *K*/*S* presents color strength, L* presents luminosity, a* presents redness-greenness (+value = red, −value = green), b* presents yellowness-blueness (+value = yellow, −value = blue), C* presents saturation, and h* presents metric hue angle [24,25].

It was evident that the *K*/*S* values of the treated cotton fabrics were higher than those of untreated cotton fabrics, and the L* values of the treated cotton fabrics were lower than those of untreated cotton fabrics, signifying that the darker color strength were obtained for the treated cotton fabrics. a* refers to the redness (+) and greenness (−), b* to the yellowness (+), and blueness (–), C* to the color saturation, and *h°* to the hue [24,25]. Both the a* and b* of the treated cotton fabrics were negative and the absolute values were higher than those of untreated cotton fabrics, which indicated that the printing color of the treated cotton fabrics became more greenness and blueness. The C* values of the treated cotton fabrics were larger than those of untreated cotton, indicating that the printing color of the treated cotton fabrics were brighter. The *h°* values of untreated and treated fabrics were close, i.e., the nanospheres modification process did not change the color hue.

The rubbing and washing color fastness of ink-jet printed cotton fabrics with different structures under the optimal ink-jet printing condition are depicted in Table 3. It was apparent that the ink-jet printed cotton fabrics with different structures exhibited outstanding rubbing fastness and washing fastness, indicating that the modification process does not affect color fastness of cotton fabrics.

**Figure 7:**
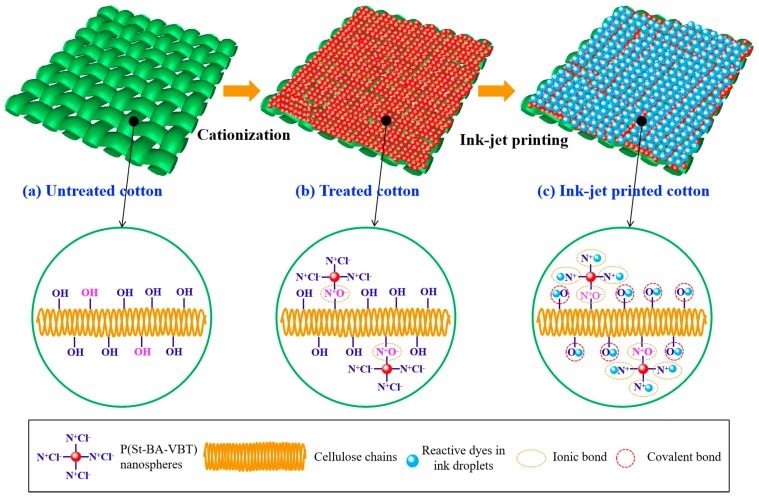
The mechanism of nanospheres modification and ink-jet printing of cotton fabrics.

Figure 7 shows the mechanism of P(St-BA-VBT) nanospheres modification and ink-jet printing of cotton fabrics. Some cellulose hydroxyl groups (Cell-OH) were converted into cellulose anions (Cell-O^-^) when the cotton fabric was modified with an alkaline solution, as shown in Figure 7a. The P(St-BA-VBT) nanospheres could be spontaneously adsorbed on the surfaces of cotton fiber through the electrostatic attraction [17] between the ammonium salt ions (-N^+^(CH_3_)_3_) and cellulose anions (Cell-O^-^) in the nanospheres modification process. Consequently, the positive charge bonding sites on the cotton fiber surfaces were introduced owing to the presence of quaternary ammonium groups, as shown in Figure 7b. Furthermore, there are two chemical bonds between the reactive dyes and the cationic cotton fibers in the inkjet printing process, which are covalent bonds and ionic bonds, respectively, as shown in Figure 7c. Because the reactive dye molecules not only reacted with the cotton hydroxyl groups but also reacted with the quaternary ammonium groups of the nanospheres in the ink-jet printing process, resulting in the short steam time, lower alkali consumption, higher color strength, and better pattern sharpness.

**Figure 8:**
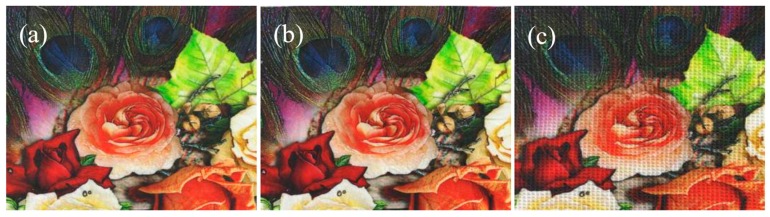
Scanned images of ink-jet printed plain fabrics (**a**), twill fabrics (**b**), and honeycomb fabrics (**c**). All fabrics were padded through modification fluid (1 g/L of nanospheres and 10 g/L of sodium bicarbonate) steamed at 102 ± 1 °C for 6 min.

As described above, the nanosphere cationization modification of cotton fabrics can avoid the use of urea, with the advantage of short steam time, lower alkali consumption, higher color strength, and better pattern sharpness [36], as we expected in Figure 8.

### 3.6. SEM Analysis of Cotton Fabrics

**Figure 9:**
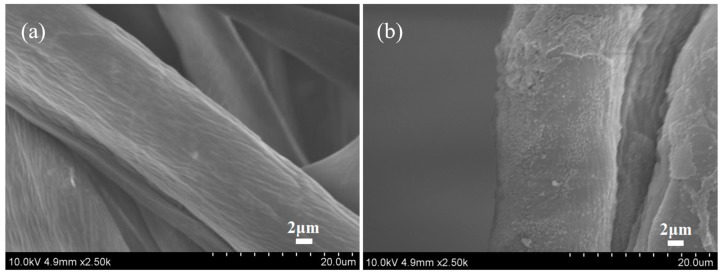
SEM images of untreated cotton fabrics (**a**) and treated cotton fabrics (**b**).

The SEM images in Figure 9 show the morphology of the untreated and treated cotton fabrics. It was apparent that untreated cotton fabrics displayed many cracks and grooves. The treated cotton fabrics showed that a large number of nanospheres were deposited on the surface of the fiber, and the nanospheres on the fiber surfaces did not form a continuous film and were randomly distributed, similar to the previous study [27,37]. Besides, the size of the nanospheres was significantly smaller than the diameter of the cotton fibers, indicating that the cationization of cotton fabrics had little effects on the spread and diffusion of reactive dye ink droplets on the surfaces of fabrics or fibers [15]. Compared to the previously study [18], the cationization of cotton fabrics modified with nanospheres is an effective method for improving the color performance of reactive dyes ink-jet printing.

### 3.7. XPS Analysis of Cotton Fabrics

**Figure 10:**
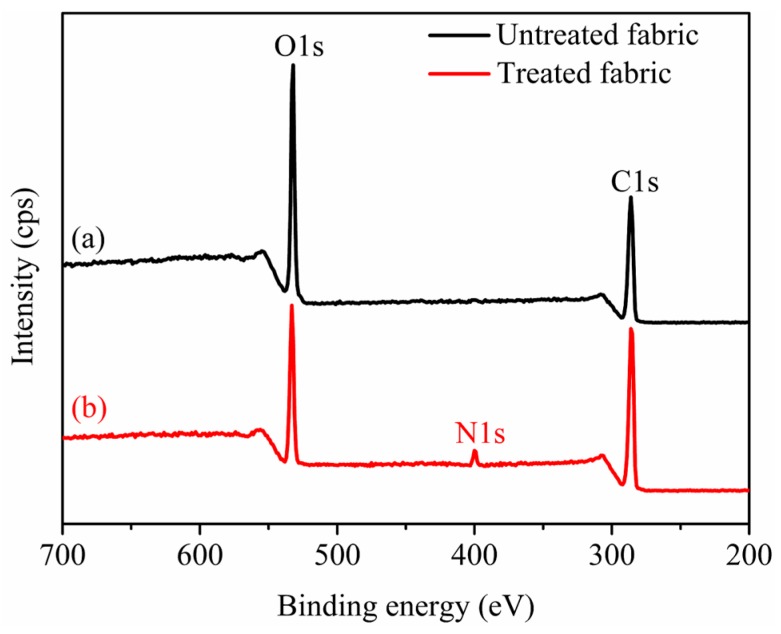
XPS spectrums of untreated cotton fabrics (**a**) and treated cotton fabrics (**b**).

As shown in Figure 10, the characteristic peaks of carbon (C), oxygen (O), and nitrogen (N) atoms can be clearly observed. There were C1s and O1s peaks at 286.18 eV and 533.08 eV of untreated and treated cotton fabrics [38], respectively. Whereas the treated cotton fabrics displayed the new peaks of N1s peak at 399.89 eV, which were indicative of the presence of nanospheres adsorbed on the fiber surfaces. Table 4 showed the atomic composition of untreated and treated cotton fabrics. Compared to untreated fabrics, the treated fabrics increased the carbon content and nitrogen content by 1.51% and 3.07%, respectively, and decreased the oxygen content by 12.37%. Besides, the treated fabrics had lower O/C atomic ratio (0.43) than the untreated cotton fabrics (0.49) [39]. The nitrogen content of the treated fabrics increased owing to the presence of nanospheres adsorbed on the fiber surfaces which correspond to N elements of quaternary ammonium group (-N^+^(CH_3_)_3_) as shown in Figure 2.

### 3.8. FTIR Analysis of Cotton Fabrics

**Figure 11:**
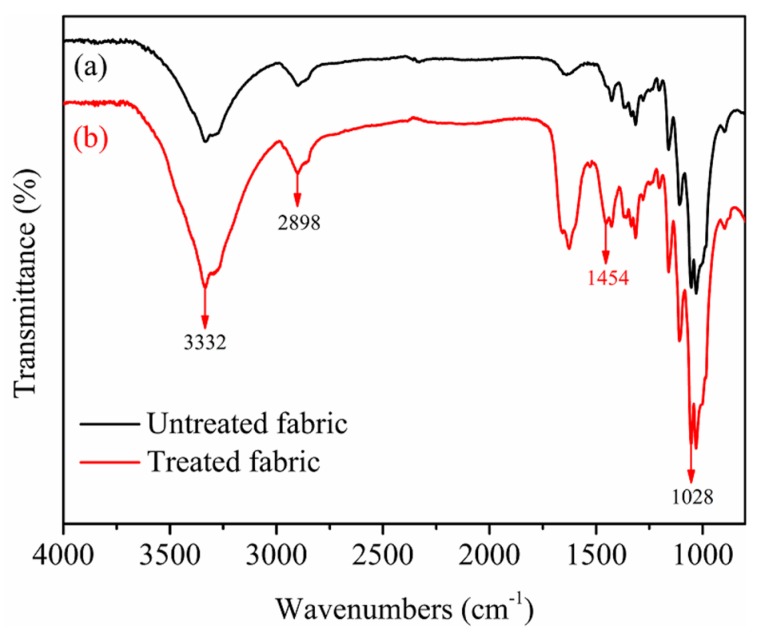
FTIR spectrums of untreated cotton fabrics (**a**) and treated cotton fabrics (**b**).

Figure 11 depicts the FTIR spectrums of untreated and treated cotton fabrics. It was observed that the strong and broad bands located around 3332 cm^−1^ characteristic to the O–H stretching vibrations occurred in both fabrics untreated and treated by nanospheres. The band located at 2898 cm^−1^ in both fabrics untreated and treated by nanospheres was assigned to the C-H stretching vibration [40]. The absorption at 1028 cm^−1^ in both fabrics untreated and treated by nanospheres can be ascribed to the C-O stretching deformation. By comparing the spectrums and data of untreated and treated fabrics, a new absorption peak occurred at 1454 cm^−1^ [41] can be attributed to the stretching vibration of C-N in quaternary ammonium group N^+^(CH_3_)_3_ of cationic P(St-BA-VBT) nanospheres, indicating the presence of cationic nanospheres on the surface of cotton fiber [17].

### 3.9. Tensile Breaking Strength of Cotton Fabrics

**Figure 12:**
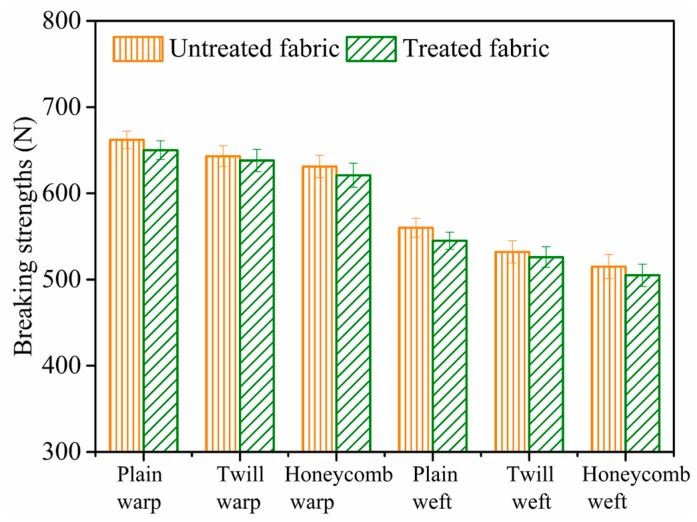
Tensile breaking strength of untreated and treated cotton fabrics.

Tensile breaking strength of untreated and treated cotton fabrics with different structures was depicted in Figure 12. It was apparent that the tensile breaking strength of treated cotton fabrics under the nanosphere concentration of 1 g/L was almost the same with the untreated fabrics. The error bars are standard deviations from three independent measurements. The results indicated that nanospheres modification process of the cotton fabrics did not affect the tensile breaking strength of the cotton fabrics [15].

## 4. Conclusions

In this study, a cationic P(St-BA-VBT) nanospheres with positive charges was synthesized by soap-free emulsion polymerization. After the pad-cure process, the nanospheres were deposited on the surface of the modified cotton fibers successfully without forming a continuous film structure. XPS and FTIR results demonstrated that P(St-BA-VBT) nanosphere was adsorbed on the surface of cotton fibers successfully. The results showed that plain, twill and honeycomb fabrics displayed excellent color strength and outline sharpness when cationic P(St-BA-VBT) nanospheres concentration was 1 g/L, sodium bicarbonate was 10 g/L, and steaming time was 6 min. It was found that plain fabrics exhibited the smallest *K*/*S* values, and the best outline sharpness, followed by twill fabrics, and honeycomb fabrics displayed the largest *K*/*S* values and the worst outline sharpness under the same conditions. In addition, the three fabrics exhibited outstanding rubbing fastness and washing fastness. And the tensile breaking strength of the cotton fabrics was almost the same before and after treated with the nanospheres. Therefore, the P(St-BA-VBT) nanospheres modification of the cotton fabrics provides a novel potential approach to improve color strength, pattern sharpness and colorfastness without using urea resulting in better quality, lower costs, and lesser harm to the environment.

## Figures and Tables

**Table 1:** Roughness, thickness and porosity of fabrics with different structures.

Fabrics	Warp Roughness (μm)	Weft Roughness (μm)	Thickness (mm)	Porosity (%)
Plain	2.8	5.2	0.6	84.5
Twill	3.5	6.2	0.8	85.5
Honeycomb	5.7	8.4	1.7	86.6

**Table 2:** The color strength and colorimetric parameters of ink-jet printed cotton fabrics with different structures *^a^*.

Cotton Fabric	Weave	Color Characteristic Values					
		*K*/*S*	L*	a*	b*	C*	*h*°
Untreated	Plain	16.6	56.9	−32.7	−29.8	44.2	222.4
	Twill	17.9	55.9	−33.3	−31.9	46.1	223.8
	Honeycomb	18.8	55.4	−34.1	−32.3	47.0	223.4
Treated	Plain	20.2	54.5	−33.5	−32.3	46.5	223.9
	Twill	21.4	53.4	−34.3	−33.3	47.8	224.1
	Honeycomb	22.8	52.6	−34.1	−33.4	47.7	224.4

*^a^* The fabrics were printed at a resolution of 720 × 720 dpi and a cyan color coverage of 100%. The printed fabrics were steamed at (102 ± 1 °C for 6 min with saturated steam.

**Table 3:** Color fastness of ink-jet printed cotton fabrics with different structures.

Cotton Fabric	Weave	Rubbing Fastness		Washing Fastness		
		Dry	Wet	SW	CC	SC
Untreated	Plain	4–5	3	4–5	4–5	4–5
	Twill	4–5	3–4	5	5	5
	Honeycomb	4–5	4	5	5	5
Treated	Plain	4–5	3–4	4–5	4–5	4–5
	Twill	4–5	3–4	5	5	5
	Honeycomb	4–5	4	5	5	5

Color change (CC), Staining on cotton fabric (SC), Staining on wool fabric (SW).

**Table 4:** Chemical composition of untreated and treated cotton fabrics.

Cotton Fabric	Binding Energy (eV)			Atomic Concentration (%)			Atomic Ratio O/C
	C1s	O1s	N1s	C1s	O1s	N1s	
Untreated	286	533	400	67.01	32.99	-	0.49
Treated				68.02	28.91	3.07	0.43
